# COVID-19 Management and Arrhythmia: Risks and Challenges for Clinicians Treating Patients Affected by SARS-CoV-2

**DOI:** 10.3389/fcvm.2020.00085

**Published:** 2020-05-05

**Authors:** Alexander Carpenter, Owen J. Chambers, Aziza El Harchi, Richard Bond, Oliver Hanington, Stephen C. Harmer, Jules C. Hancox, Andrew F. James

**Affiliations:** ^1^School of Physiology, Pharmacology and Neuroscience, University of Bristol, Bristol, United Kingdom; ^2^Liverpool Heart and Chest Hospital NHS Foundation Trust, Liverpool, United Kingdom; ^3^Gloucestershire Hospitals NHS Foundation Trust, Gloucester, United Kingdom

**Keywords:** COVID-19, QT, arrhythmia, hERG, drug safety, QTc

## Abstract

The COVID-19 pandemic is an unprecedented challenge and will require novel therapeutic strategies. Affected patients are likely to be at risk of arrhythmia due to underlying comorbidities, polypharmacy and the disease process. Importantly, a number of the medications likely to receive significant use can themselves, particularly in combination, be pro-arrhythmic. Drug-induced prolongation of the QT interval is primarily caused by inhibition of the hERG potassium channel either directly and/or by impaired channel trafficking. Concurrent use of multiple hERG-blocking drugs may have a synergistic rather than additive effect which, in addition to any pre-existing polypharmacy, critical illness or electrolyte imbalance, may significantly increase the risk of arrhythmia and Torsades de Pointes. Knowledge of these risks will allow informed decisions regarding appropriate therapeutics and monitoring to keep our patients safe.

## Introduction

The novel coronavirus disease of 2019 (COVID-19) pandemic is uncharted territory for clinicians and healthcare systems alike. The potential for a high volume of severely unwell patients creates a significant challenge. In particular, the combination of severe infection, respiratory dysfunction, sepsis, shock, and/or haemodynamic instability (1–3) present significant potential for myocardial injury and potentially dangerous arrhythmia. Not only do these pathophysiological states promote the formation of arrhythmia, but unstable arrhythmia could present a significant threat to this cohort of patients who are at risk of haemodynamic instability.

Due to the potential strain on healthcare systems, it is likely that non-specialists will be involved in the management of COVID-19 patients including those experiencing arrhythmia. Similarly, clinicians may face situations where they are using multiple concurrent medications with which they may not be entirely familiar. Here, we attempt to summarize how the use of multiple different types of medication could contribute (synergistically, in some instances) to increase arrhythmic risk. In particular, we focus on the risk of drug-induced QT interval prolongation (commonly referred to as acquired long QT syndrome; aLQTS) which is a serious issue for many of the medications which are likely to be used.

While the use of drugs known to prolong the QT interval may well be necessary and unavoidable, an awareness and understanding of this risk should guide additional safety measures such as monitoring of the corrected QT interval (QTc) on 12-lead electrocardiogram (ECG) measurement. For cases of a prolonged QT interval there is a risk, particularly with a rate-corrected QT interval (QTc) of >500 ms (4), of dangerous arrhythmia including Torsades de Pointes (TdP); close monitoring and involvement of specialist cardiology input should be sought, as well as minimizing, where possible, the use of other known QT-prolonging medication.

## Increased Baseline Arrhythmic Risk in COVID-19 Patients

From the data available, COVID-19 seems to cause more serious disease in older patients and those with comorbidities (1–3, 5–7). Zhou et al. (3) described hypertension, diabetes and coronary heart disease as the three most prevalent comorbidities in COVID-19 patients from two hospitals at the epicenter of the initial outbreak in Wuhan. Not only were these comorbidities associated with a significantly worse outcome, they are also, in combination with advanced age, significant risk factors for arrhythmia. Increasing age and co-morbidities also increase the likelihood of pre-existing polypharmacy, which may prove problematic in the context of additional potentially QT-prolonging medication. Unfortunately, published data of COVID-19 patient cohorts to date do not seem to include any ECG or specific arrhythmia data, although no doubt we will gain a better picture as our understanding of the disease and its management evolves.

## Increased Arrhythmic Risk of COVID-19 Infection

SARS-CoV-2 is thought to gain entry to human host cells via the angiotensin converting enzyme 2 (ACE2) receptor, which is highly expressed in the heart and lungs and to which the viral spike protein has high affinity (8, 9). Interestingly, data during the previous SARS-CoV outbreak demonstrated ACE2-dependent cardiac infection and inflammation in both mouse and human hearts (10). The down-regulation of affected ACE2 receptors was suggested as a potential contributory factor to SARS-associated myocarditis and subsequent cardiomyopathy, with inflammation and fibrosis likely to provide a substrate for arrhythmia.

The risk of arrhythmia is likely to increase with the development of significant infection, and increase as the severity of the infection and/or systemic inflammatory response increases (11). Significant myocardial damage and fulminant myocarditis have been described (2, 3, 5), and cardiac arrest associated with ventricular arrhythmia (as well as non-shockable rhythms) is reported (7). Du et al. reported some form of arrhythmia present in 60% of a group of fatal cases, with cardiac arrest or malignant arrhythmia listed as the cause of death for over 10% of cases (12).

There is increasing awareness of the development in severely unwell patients of a hyperinflammatory state or cytokine storm (13) which can lead to multi-organ failure. Recent work strongly suggests this hypercytokinemia (in particular elevated levels of interleukin-6) further increases arrhythmic risk via multiple mechanisms, including, notably, hERG blockade (14) and QT prolongation (15, 16). Myocarditis itself is a heterogenous condition associated with a number of arrhythmic states including bradyarrhythmia and atrial or ventricular tachyarrhythmia (17). Data are urgently needed to describe the unique arrhythmic challenges of COVID-19 myocarditis.

Additionally, the multiple medications likely to receive significant use (antibiotics, anesthetic agents, anti-arrhythmic agents, and potentially specific agents to target COVID-19 such as anti-malarial or anti-viral medications) may indeed contribute to a pro-arrhythmic state in a patient group already at risk. Importantly, the additional risk of QT prolongation with some potential combinations of these medications may be synergistic rather than simply additive, due largely to their unique mechanisms of ion channel blockade. Finally, significant electrolyte disturbances, common in unwell patients, will further exacerbate arrhythmic risk (18).

## Clinical Management of COVID-19: Significant Potential for hERG Blockade and QT Prolongation

The hERG (or “Kv11.1”) potassium channel (encoded by *human Ether-à-go-go Related Gene;* alternative nomenclature *KCNH2*) mediates the rapid component of cardiac delayed rectifier K^+^ current (also known as I_Kr_). Briefly, this channel is crucial to the repolarization phase of the cardiac action potential; we recommend an excellent review article (19). Inhibition of hERG is considered to be the most common and important mechanism of drug-induced QT prolongation (20, 21) and occurs through direct pharmacological channel block and/or impaired trafficking of hERG channels to the cell membrane. Consequently, hERG testing is a requirement during novel drug development: indeed, prolongation of the QT interval (and its association with dangerous TdP) has been the biggest cause of restriction or withdrawal of drugs already on the market (21–23).

Importantly, recent data including work from our group suggests that the effect on hERG (and thus on QT-prolongation) of multiple drugs may not be simply additive but synergistic (24, 25) (i.e., an effect in excess of the sum of their individual parts). This is particularly relevant when considering an older patient group who may already be taking hERG-blocking medication. In particular, the antimalarial medications chloroquine and hydroxychloroquine (26, 27) are receiving particular attention as antiviral agents: these drugs are multichannel blockers with particular effects on hERG and Kir2.1 and are likely to cause significant QT prolongation at the concentrations effective against SARS-CoV-2 *in vitro* (28–30), especially when combined with other antivirals (such as lopinavir/ritonavir) or antibiotics (macrolides and fluroquinolones being particularly notable) (26). Further information should be sought regarding novel antiviral agents (31) currently undergoing clinical trials; for example, favipiravir has been associated with QT interval prolongation in a case report (32) and remdesivir awaits comprehensive testing.

Combination therapy with azithromycin and hydroxychloroquine is undergoing testing at present (33, 34). Notably, part of a Brazilian study comparing low vs. high dose chloroquine, in combination with ceftriaxone and azithromycin with or without oseltamivir, was terminated early due to safety concerns; with 25% of those in the high-dose arm showing QT prolongation (vs. 11% in the low-dose arm) and two patients in the high-dose arm experiencing ventricular tachycardia prior to death (35). Similarly, the head of a French cardiology unit reported they had prematurely terminated their hydroxychloroquine-azithromycin COVID-19 trial due to unacceptable QT prolongation (36). The randomized DisCoVeRy (NCT04315948), SOLIDARITY (EudraCT Number 2020-000982-18), and RECOVERY (EudraCT Number 2020-001113-21) studies will provide important evidence regarding the effectiveness and safety of various antiviral and antimalarial drugs in the treatment of COVID-19 patients (37).

Online resources can be consulted to clarify which drugs are associated with QT prolongation (see www.crediblemeds.org). Table 1 lists medications associated with QT prolongation which may be commonly used in the management of COVID-19 [as taken from the updated WHO management guidance (38) with additions from authors' clinical experience from UK practice]. The list is not exhaustive but attempts to highlight potential areas of risk when using these medications, as well as their use in combination.

**Table 1:** A list of medications which could be used in the management of COVID-19 which are also associated with risk of QT prolongation.

**Type of medication**	**Drugs**
Anesthetic agents	Propofol, sevoflurane
Antibiotic, antiviral or antifungal medication	Macrolides, fluoroquinolones, fluconazole, pentamidine, lopinavir/ritonavir^*^, favipiravir^*^
Anti-emetics	Domperidone, levomepromazine, ondansetron
Anti-arrhythmics	Amiodarone, flecainide, ibutilide, procainamide, quinidine, sotalol
Anti-psychotics (used for delirium)	Haloperidol, quetiapine, risperidone
Other potential therapies under consideration	Antimalarials such as chloroquine, hydroxychloroquine or mefloquine

*List intended to be illustrative as to risks and is not exhaustive. Further up to date information can be found online from trusted sources (www.crediblemeds.org)*.

* *possible rather than established risk*.

## Discussion

The recent publication by Ackerman et al. of urgent guidance and a practical flow-chart regarding safe use of QT-prolonging medication is very welcome, and should be consulted as an aid to manage risk in the setting of QT prolongation (39), as should the Heart Rhythm Society (HRS) Task Force update (40). Other resources provide valuable guidance for clinicians dealing with specific patient populations: those with congenital heart disease (41) or inherited arrhythmia syndromes (42). Of note, a case report has reported significant QT prolongation (620 ms) in a patient with COVID-19 treated with multiple hERG blocking medications (including levofloxacin, hydroxychloroquine and azithromycin), which was successfully managed with drug cessation and intravenous lignocaine (43). Separately, mexiletine has also been suggested to be effective in treating TdP associated with acquired long QT syndrome (44).

COVID-19 represents a step into the unknown: not only are we grappling to come to terms with effective management of this new disease, but so too with safe use of these treatments. Effective therapy will be welcome, but the use of multiple drugs in combination has to be exercised with caution as it may increase the risk of QT prolongation and Torsades de Pointes, largely via pharmacological hERG blockade. Knowledge of this risk enables clinicians to ensure adequate monitoring of the QT interval and management of arrhythmic risk, maximizing safety for our patients in this challenging time.

## Author Contributions

All authors contributed to the concept, planning and writing of this mini-review, and approved the manuscript prior to submission.

## Conflict of Interest

The handling editor is currently organizing a Research Topic with one of the authors AJ, and confirms the absence of any other collaboration. The remaining authors declare that the research was conducted in the absence of any commercial or financial relationships that could be construed as a potential conflict of interest.
